# Epidemiology of forest malaria in Central Vietnam: the hidden parasite reservoir

**DOI:** 10.1186/s12936-015-0601-y

**Published:** 2015-02-19

**Authors:** Pham Vinh Thanh, Nguyen Van Hong, Nguyen Van Van, Carine Van Malderen, Valérie Obsomer, Anna Rosanas-Urgell, Koen Peeters Grietens, Nguyen Xuan Xa, Germana Bancone, Nongnud Chowwiwat, Tran Thanh Duong, Umberto D’Alessandro, Niko Speybroeck, Annette Erhart

**Affiliations:** National Institute of Malariology, Parasitology and Entomology (NIMPE), Hanoi, Vietnam; Provincial Malaria Station, Tam Ky City, Quang Nam Province Vietnam; Université Catholique de Louvain (UCL), Brussels, Belgium; Université Ccatholique de Louvain (UCL), Louvain-la-Neuve, Belgium; Institute of Tropical Medicine Prince Leopold (ITM), Antwerp, Belgium; Shoklo Malaria Research Unit, Mae Sot, Tak Province Thailand; Medical Research Council Unit (MRC Unit), Fajara, The Gambia

**Keywords:** Malaria, Sub-patent infections, Elimination, Central Vietnam

## Abstract

**Background:**

After successfully reducing the malaria burden to pre-elimination levels over the past two decades, the national malaria programme in Vietnam has recently switched from control to elimination. However, in forested areas of Central Vietnam malaria elimination is likely to be jeopardized by the high occurrence of asymptomatic and submicroscopic infections as shown by previous reports. This paper presents the results of a malaria survey carried out in a remote forested area of Central Vietnam where we evaluated malaria prevalence and risk factors for infection.

**Methods:**

After a full census (four study villages = 1,810 inhabitants), the study population was screened for malaria infections by standard microscopy and, if needed, treated according to national guidelines. An additional blood sample on filter paper was also taken in a random sample of the population for later polymerase chain reaction (PCR) and more accurate estimation of the actual burden of malaria infections. The risk factor analysis for malaria infections was done using survey multivariate logistic regression as well as the classification and regression tree method (CART).

**Results:**

A total of 1,450 individuals were screened. Malaria prevalence by microscopy was 7.8% (ranging from 3.9 to 10.9% across villages) mostly *Plasmodium falciparum* (81.4%) or *Plasmodium vivax* (17.7%) mono-infections; a large majority (69.9%) was asymptomatic. By PCR, the prevalence was estimated at 22.6% (ranging from 16.4 to 42.5%) with a higher proportion of *P. vivax* mono-infections (43.2%). The proportion of sub-patent infections increased with increasing age and with decreasing prevalence across villages. The main risk factors were young age, village, house structure, and absence of bed net.

**Conclusion:**

This study confirmed that in Central Vietnam a substantial part of the human malaria reservoir is hidden. Additional studies are urgently needed to assess the contribution of this hidden reservoir to the maintenance of malaria transmission. Such evidence will be crucial for guiding elimination strategies.

## Background

The past 20 years of continued malaria control efforts have resulted in the elimination of this disease in several provinces of Northern and Southern Vietnam [1]. In 2011, the Vietnamese Government officially launched the National Malaria Control and Elimination Programme aiming at malaria elimination for the whole country by 2030 [2]. However, such an ambitious goal faces several challenges that include forest malaria, seasonal population movements (internal and across international borders) and emerging drug resistance.

Currently, most malaria morbidity (18,387 confirmed cases in 2012) and malaria deaths (eight in 2012) occur in 21 out of 58 provinces (=25% of total population) and are located in Central and Central-Southern Vietnam [3], where standard vector control interventions are unable to interrupt forest malaria transmission and where *Plasmodium falciparum* resistance to artemisinin derivatives has been reported [4]. Asymptomatic malaria infections are common in remote and forested areas of Central Vietnam [5-7], particularly among local ethnic minorities in which the burden of *Plasmodium vivax* is particularly high. A recent survey carried out in Ninh Thuan Province (Central-Southern Vietnam), in which filter paper blood samples were analysed by molecular techniques, showed the presence of a largely hidden human reservoir of malaria infections with numerous sub-patent infections (detected only by PCR but not by microscopy) including mixed infections with *Plasmodium malariae* and *Plasmodium ovale* [8]. Besides the difficulty of identification by standard microscopy, *P. vivax* and *P. ovale* may have dormant liver forms (hypnozoites) that can reactivate at varying times after the primary infection. Vietnamese treatment guidelines recommend the use of both a three-day course of chloroquine (0.25 mg/kg) and a 14-day course of primaquine (0.25 mg/kg/day) to clear both peripheral blood and liver stages of infection. However, a 14-day course of primaquine is rarely followed due to fears of haemolysis in glucose-6-phosphate dehydrogenase deficient (G6PDd) individuals [9]. In addition, compliance to the 14-day primaquine treatment is usually low. The latter issue was addressed by recommending in January 2007 (decision number 339/QĐ-BYT) a shorter but higher dosage of primaquine, i.e., ten days at a daily dose of 0.5 mg/kg. A cohort study was set up in Quang Nam Province from 2009 to 2011 to assess the short- and long-term efficacy of the new regimen. The present paper reports the baseline malaria prevalence and related risk factors among the study population before the start of the cohort.

## Methods

### Study site and population

**Figure 1:**
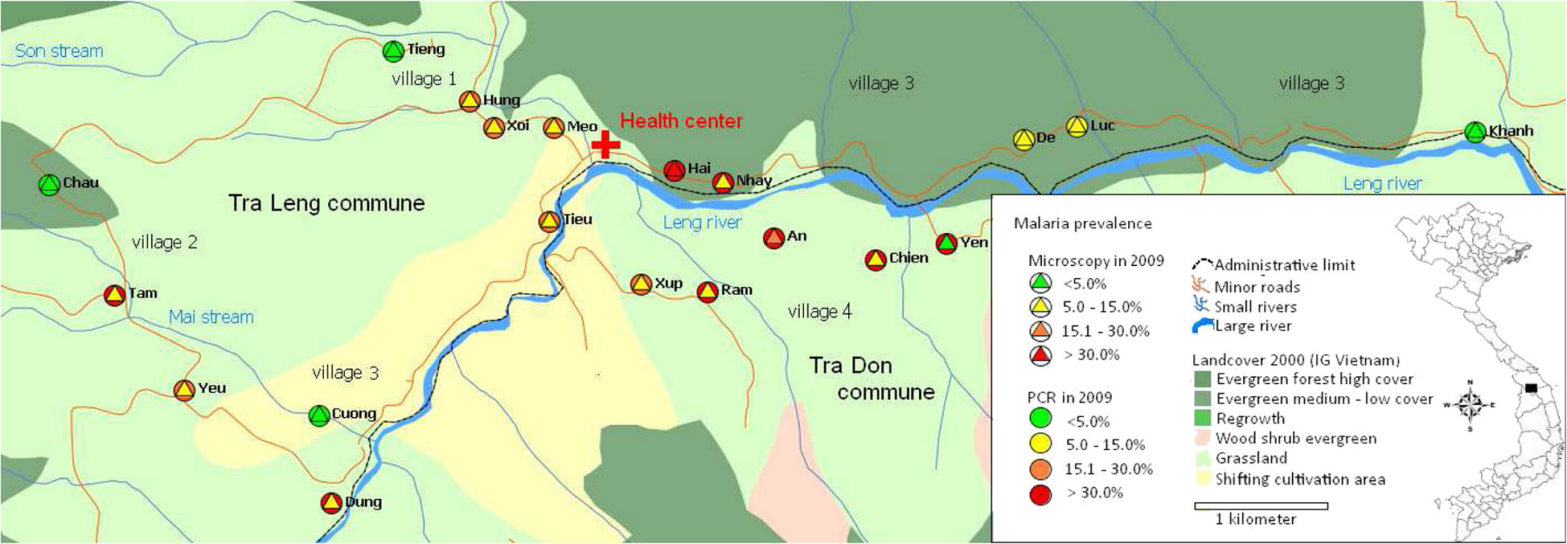
**Map of the study area showing malaria prevalence in the 20 study clusters (by microscopy and PCR).**

The study was carried out in four villages located in two communes (Tra Leng and Tra Don) situated in Nam Tra My district in Quang Nam Province (Central Vietnam) (Figure 1). Study villages were located in a remote forested valley accessible only on foot (five hours) or motorbike (two hours) on a mountain track from the nearest health centre in Tra Don commune. Villages were extremely scattered, with households grouped in clusters of four to 45 houses situated at variable distance from each other. The number of clusters varied by village with four clusters in Village 1, two in Village 2, nine in Village 3, and five in Village 4. All study clusters were served only by the CHC in Tra Leng since the one in Tra Don commune was too far. Village 3 and 1 were located along the way to and around the CHC, respectively, while Villages 2 and 4 were situated at 4- and 3 hours walking distance (for the farthest clusters) from the CHC. In addition, there was a river between the centre of the commune and Village 4 whose access was almost impossible during the heavy rains.

The population mainly belonged to the M’nong and Ca Dong ethnic groups living in very poor socio-economic conditions, mainly subsistence farming, practising slash-and-burn agriculture in forest fields with maize, manioc and rice. Malaria transmission is perennial with two peaks, one in May-June and the other in October-November, with the two main vectors species being *Anopheles dirus sensu stricto* and *Anopheles minimus sensu stricto* [10,11]. Malaria control activities are based on free-of-charge, early diagnosis and treatment with an artemisinin-based combination (ACT; dihydroartemisinin-piperaquine) and regular indoor residual spraying (IRS; alpha-cypermethrin) as bed net use was not very popular in the study area at the time of the survey (Nguyen Van Van, personal communication).

The Commune Health Centre (CHC) located in the centre of the commune (Village 1) was hardly accessible for Village 4 during the rainy season because of heavy rains and flooding. The local health staff (one midwife, three nurses, one microscopist, and one pharmacist) provided free-of-charge health care with the support of village health workers (VHWs).

### Data collection

In February 2009 a full census of the study population (1,810 individuals) was carried out to collect household as well as individual socio-demographic data (gender, age, location, occupation, assets, distance to the fields, number of available bed nets per household, housing structure, etc.). Each resident in the study area was allocated a unique ID code. Each house was mapped using a geography position system device (Garmin eTrex Legend HCx Personal Navigator) [12].

In April 2009, the entire study population was screened for *P.vivax* infections to identify potential study participants for a cohort to be followed prospectively. This started by informing first all commune, village and household leaders on the objectives and study procedures and then the individual study subjects, who were all invited to be screened after oral informed consent. During the screening, each participant was interviewed for previous malaria symptoms during the previous 48 hours, the axillary temperature was checked and a blood slide collected for light microscopy (LM). Confirmed malaria infections were treated according to the national treatment guidelines.

In addition to blood slides taken during the screening, an additional blood sample was taken for haemoglobin measurement and for later molecular analysis (PCR) in a random sample of study participants (n = 327). This was done by randomly choosing one individual in each household after blindly drawing an ID number among those allocated to the house during the census. If the selected subject was temporarily absent, the survey team would return later; however, if the subject was absent for a long time or not willing to participate, a second drawing would be done. Survey participants (i.e., with additional blood samples) were asked to give their written informed consent (parent/guardian for children) after being explained the purpose of the additional sampling and investigations. Among these subjects, a face-to-face interview was done to collect data on the different outdoor activities in and outside the community, sleeping habits, as well as malaria prevention measures. For children under 12 years old, the parent/guardian would answer the questions.

### Laboratory procedures

Thick and thin films were stained with a 3% Giemsa solution for 45 minutes. The number of asexual forms per 200 white blood cells (WBCs) was counted and parasite densities were computed assuming a mean WBCs count of 8,000/μl. Gametocytes were also counted. A slide was declared negative when no parasite was seen after counting 1,000 WBCs. All slides were read independently by two expert microscopists. In case of discrepant results, they re-examined the slide together until agreement was reached. Quality control of blood slides was done on all positives and 10% of negative blood slides by a senior laboratory technician at the National Institute of Malariology, Parasitology and Entomology (NIMPE), Hanoi; in case of disagreement, a second senior technician would re-read the slide until an agreement was reached.

Haemoglobin concentration was measured with the HemoCue Hb 301 device following the manufacturer’s instructions [13]. Filter paper blood samples (FPBS) were dried outside in direct sunlight and kept in individual, sealed, plastic bags containing silica gel. All FPBS were stored at 4°C in the CHC refrigerator before being shipped to NIMPE, Hanoi, where they were kept at −20°C. DNA extraction was done using the QIAamp DNA Micro Kit (Qiagen, Hilden Germany), and a species-specific, semi-nested, multiplex PCR (SnM-PCR) was performed to detect *P. falciparum, P. vivax, P. malariae,* and *P. ovale* [8]. The PCR products (5 μl) were subjected to electrophoresis on a 2% agarose gel in 0.5X TAE buffer for 90 minutes at 100 V. The gels were stained with ethidium bromide and visualized with ultraviolet light. The sizes of the PCR products were compared with a standard 100-basepair DNA ladder (Fermentas, Burlington, Ontario, Canada) and positive controls of each *Plasmodium* species. Cross-contamination during handling was checked for by implementing negative controls in each step from extraction to the nested PCR step. Quality control was done on 10% of the samples for which the SnM-PCR was repeated blindly by a senior technician. In case of discrepancy, the sample was re-analysed until agreement was reached.

All survey samples were analysed for G6PD deficiency at the Shoklo Malaria Research Unit, by genotyping for the Viangchan mutation following a modified protocol published by Nuchprayoon *et al*. [14]. DNA was extracted using the Saponin-Chelex method [15]. Genotyping for the Viangchan mutation (871G > A) was performed by PCR/RFLP method using published primers [14] and MyTAq™ DNA polymerase (Bioline, UK) with the following amplification conditions: initial denaturating step at 95°C (5 min) followed by 30 cycles of 95°C (30 sec), 57°C (20 sec), and 72°C (15 sec) and final elongation step at 72°C for 7 min. Amplified fragments were digested with XbaI enzyme and visualized on a 3% agarose-nusieve gel. Quality control was performed on 10% of randomly selected survey samples; in case of disagreement, the sample was re-analysed by another senior technician.

### Data management and statistical analysis

Sample size: According to the provincial malaria station data on surveys carried out in April-May, the overall parasite rate was around 16% (ranging from 5 to 39% across hamlets) and the prevalence of *P. vivax* at 9%. The sample size was calculated by assuming a minimal prevalence of 9%, with 3% precision at 5% significance level and adding 10% security margin; a total of 330 individuals were needed for the survey (“CSample” command/EpiInfo6). Therefore, to simplify sampling procedures, one individual in each house visited during screening was randomly selected to be included in the survey.

Data were double-entered and cleaned using Epidata version 3.1 free software [16]. The data set was analysed using STATA version 11 (Stata Corp, College Station, TX, USA). Descriptive statistics were used to compute baseline socio-demographic characteristics as well as malariometric indices by village, and significant differences were tested for using either a Chi-square test or Student *t*-test as required, and a p-value <0.05 was used as cut-off for significance.

Three different variables for livestock ownership (number of i) buffaloes, ii) cows, and iii) pigs) were considered as the best proxy (after discussion with household leaders) for the economic status of the households as all inhabitants were subsistence farmers and generally poor. In order to aggregate multiple variables to a single measure of economic status, a principal component analysis was performed [17]. Using the factor scores from the first principal component as weights, an index was created for the economic status of each household then the index were categorized by dividing the score into tertile.

The survey design (survey dataset) was taken into account using the svy- command in STATA, with villages as *strata*, and household sizes as p*-weights*. A survey logistic regression *(“svy”* command in STATA) was used to carry out a multivariate adjusted analysis for the risk of malaria infection (determined by PCR). Moreover, a classification tree analysis (CART; Salford Systems Inc, CA, USA) was performed to explore the relationship and rank the relative importance of risk factors for all malaria infections identified by PCR, as well as for patent infections only (detected both by PCR and microscopy). Sub-patent (or sub-microscopic) malaria infections are detected by PCR only. The CART analysis is a non-parametric method enabling more direct and flexible analyses since, unlike logistic regression models, it allows for co-linearity and multiple interactions between different independent variables [18]. Briefly, the building of the classification tree starts with the root node, which contains the entire set of observations. CART then finds the best possible variable to split the root node into two child nodes, by identifying the best splitting variable that maximizes the average ‘purity’ (homogeneity) of the two child nodes. To improve the stability of the CART model, a ten-fold cross-validation method was applied, and the best tree was selected by choosing the smallest tree within one standard error of the minimum error. CART also provides a ranking power of each predictor variable.

### Ethical clearance

Ethical clearance was obtained from both the ethical committee of NIMPE in Hanoi and the University of Antwerp. The fundamental principles of ethics in research on human participants were upheld throughout the project. All study participants gave their informed consent after being explained the study procedures as well as their right to withdraw without prejudice for themselves or their families.

## Results

A total of 1,810 individuals were identified during the census, representing 352 households distributed in 20 clusters within the four study villages (Table 1). Clusters varied substantially in size (range: four to 45 households) and distance from each other (Figure 1). The M’nong ethnic group, mainly living in Villages 1–3, was the most represented (79.9%), while all the Ca Dong (19.3%) lived in Village 4. The study population was young (median age = 16 years), poorly educated and with low socio-economic status. Most of the houses were provided by the government, with metal roofs (93.7%) and wooden walls (88.1%). Bed net coverage (untreated nets) was very low with more than 75% of households having no nets and only 7.7% had a sufficient number to achieve a coverage of maximum two persons/net. Most adults, both males and females, were farmers (90.5%), practising slash-and-burn agriculture in forest fields, as well as cinnamon tree cultivation; the wealthiest families had some livestock.

**Table 1:** **Baseline characteristics of the study population**

**Study population N = 1,810**	**n**	**%**
Village 1	407	22.5
Village 2	305	16.8
Village 3	751	41.5
Village 4	347	19.2
Gender		
Male	929	51.3
Female	881	48.7
Age groups		
≤9	568	31.4
10 -- 19	443	24.5
20 -- 29	321	17.7
≥30	478	26.4
Median age; [range]	16; [1; 88]	
Ethnic groups		
Cadong	350	19.3
M’nong	1,447	79.9
Kinh and others	13	0.7
Education level (age >18 years, n = 834)		
None	230	27.6
Elementary	402	48.2
Secondary and above	202	24.2
Occupation (age >18 years, n = 834)		
Students	27	3.2
Farmers	755	90.5
Others (officer, business)	52	6.2
**Households N = 352**	**n**	**%**
Average persons/household, median; [range]	5; [1; 11]	
Bed net availability		
No net	270	76.7
1-2 persons/net	21	6.0
≥3 persons/net	61	17.3
Type of roof		
Aluminium	330	93.7
Others (leaves, tiles, wood, bamboo)	22	6.3
Type of wall		
Wood	310	88.1
Bamboo	41	11.6
Brick	1	0.3
Cinnamon plantation		
Owns cinnamon plantation	332	94.3
Average walking distance (hours) from village to plantation, median; [range]	1 h. [3 min; 8 hr]	
Economic status		
Lowest income	219	62.2
Low	27	7.7
Higher	106	30.1

A total of 1,450 individuals (80.1%) were screened for malaria by LM (Table 2) and their socio-demographic characteristics were similar to those of the whole population. The main reason for non-participation was absence at the time of the screening because of schooling (pupils, students) or to field work (adults). Malaria prevalence was 7.8% (113/1,450) by LM, ranging between the four villages from 3.9 to 10.9%, and from 0 to 41.2% across the 20 clusters (Figure 1). Malaria prevalence was the highest (13.9%) in the ten to 19 years old children, except for Village 4 where the highest prevalence (15.4%) was found in younger children (<ten years).

**Table 2:** **Malariometric indices by study village (determined by microscopy and PCR)**

	**Village 1% (n)**	**Village 2**	**Village 3**	**Village 4**	**TOTAL % (n/N) [95% CI]**	
**Total screened (by microscopy)**	**350**	**256**	**532**	**312**	**1,450**	
Parasite prevalence (microscopy)	4.9 (17)	3.9 (10)	9.8 (52)	10.9 (34)	7.8 (113/1450)	[6.3; 9.6]*°*
Prevalence by age group (years):						
≤9	4.7 (6/127)	1.2 (1/84)	9.9 (20/202)	15.4 (18/117)	8.5 (45/530)	[6.3; 11.3] °°
10–19	7.6 (5/66)	7.6 (4/53)	25.6 (20/78)	11.4 (8/70)	13.9 (37/267)	[9.4; 20.0]
20–39	4.2 (4/95	5.3 (4/75)	5.6 (9/160)	7.9 (6/76)	5.7 (23/406)	[3.8; 8.3]
≥40	3.2 (2/62)	2.3 (1/44)	3.3 (3/92)	4.1 (2/49)	3.2 (8/247)	[1.6; 6.4]
Species distribution (proportion):						
*- P. falciparum*	*58.8 (10)*	*80.0 (8)*	*96.2 (50)*	*70.6 (24)*	*81.4 (92)*	[72.8; 87.8]*°*
*- P. vivax*	*41.2 (7)*	*20.0 (2)*	*1.9 (1)*	*29.4 (10)*	*17.7 (20)*	[11.6; 26.1]
*-* Mixed	*0*	*0*	*1.9 (1)*	*0*	*0.9 (1)*	[0.1; 6.3]
Parasite density (geometric mean):						
*- P. falciparum*	*1390.9*	*938.5*	*2008.3*	*3007.6*	*2006.9*	[1,523.8; 2,643.1]
*- P. vivax*	*340.9*	*423.3*	*726.6*	*794.9*	*559.7*	[301.9; 1,037.7]
Infections with gametocytes, % (n)	64.7 (11/17)	40.0 (4/10)	15.4 (8/52)	52.9 (18/34)	36.3 (41/113)	[28.0; 45.5]°°
Proportion asymptomatic, % (n)	76.5 (13/17)	80.0 (8/10)	73.1 (38/52)	58.8 (20/34)	69.9 (79/113)	[60.1; 78.2]
**Total surveyed (PCR)**	**N = 75**	**N = 55**	**N = 133**	**N = 64**	**N = 327**	
Parasite prevalence*, % (n)	16.4 (13/75)	18.9 (12/55)	20.2 (25/133)	42.5 (24/64)	23.6 (74/327)	[19.0; 28.9]
Species distribution:						
*P. falciparum (Pf)*	25.8 (4)	52.6 (6)	77.5 (17)	58.6 (14)	59.2 (41)	[47.2; 70.2]*°*
*P. vivax (Pv)*	74.2 (9)	47.4 (6)	22.5 (8)	37.9 (9)	39.6 (32)	[28.7; 51.6]
*Pf + Pv*	0	0	0	3.5 (1)	1.2 (1)	[0.2; 8.7]
Prop. sub-patent infections, % (n)	79.0 (10)	73.7 (8)	44.9 (14)	57.2 (14)	58.7 (46)	[46.2; 70.2]
Prop. asymptomatic infections, % (n)	100.0 (13)	71.9 (9)	62.3 (15)	78.6 (18)	75.4 (55)	[63.7; 84.2]
Prevalence ratio LM/PCR	0.30	0.21	0.48	0.26	0.33	
Mean haemoglobin, g/dl	13.1	13.4	11.9	11.9	12.4	**[**12.2; 12.6]**
Prevalence anaemia (Hb <10.0 g/dl)	3.4 (2)	2.7 (1)	14.9 (19)	11.7 (7)	9.6 (29)	[6.6; 13.7]*°*

°*Chi-square* p < 0.05;

*°°Chi-square* p < 0.001 (p-values are specified for significant differences between villages for each category);

* Village prevalence was estimated taking into account the survey design (weight = household size; strata = village);

** Significant Hb decrease by >1.1 g/dl in Villages 3 and 4 compared to Village 1 (svy regress p < 0.05).

Overall *P. falciparum* mono-infections represented the majority of LM detected infections (81.4%) although such proportion varied significantly between villages, i.e., from 59% in Village 1 to 96% in Village 3. The mean parasite density was significantly higher in *P. falciparum* (2,006.9/μl) than in *P. vivax* (559.7/μl) infections, and increased with increasing prevalence of infection. Most infections were asymptomatic (69.9%), and approximately one-third (36.3%) carried gametocytes, with significant differences between villages (p <0.001). The prevalence of gametocyte carriage by village was significantly correlated to the prevalence of *P. vivax* infections (R^2^ = 0.99; p = 0.002). Overall, the risk of gametocyte carriage was significantly higher in asymptomatic (46.8%) than in symptomatic (11.8%) infections (p <0.001), and in *P. vivax* (95.0%) compared to *P. falciparum* (22.8%) infections (p <0.001). In addition, the risk of asymptomatic infection was significantly higher among *P. vivax* than *P. falciparum* infections (90 *vs* 65%, p = 0.027).

**Figure 2:**
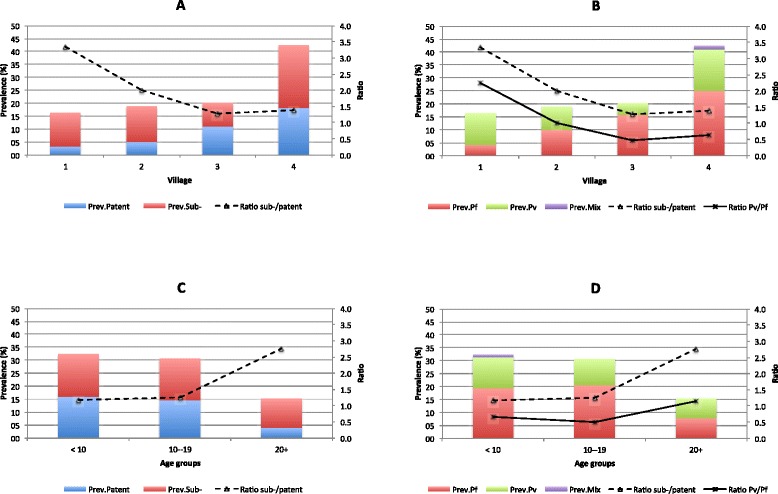
**A Ratio sub-patent/patent malaria infections according to malaria prevalence by village; B Ratio sub-patent/patent malaria infections according species prevalence by village and ratio Pv/Pf; C Ratio sub-patent infections according to prevalence by age group; D Ratio sub-/patent according to species prevalence by age group and ratio Pv/Pf.**

A total of 327 individuals were randomly selected to estimate malaria prevalence by PCR; their socio-demographic characteristics were similar to those of the whole population. Malaria prevalence estimated by PCR was about three-fold higher than by microscopy (23.6 *versus* 7.8%), ranging by village between 16.4 and 42.5% (Table 2) and by cluster between 0 and 66.7% (Figure 1). The proportion of *P. vivax* mono-infections was higher when determined by molecular methods than by LM (39.6%, 95% CI [28.7; 51.6] *vs* 17.7%, 95% CI [11.6; 26.1]), while mixed species infections remained scarce (n = 1). More than half (58.7%) of all infections were sub-patent, i.e., negative by LM, and this proportion increased with decreasing village prevalence of infection as shown by the evolution of the ratio between sub-patent and patent infections (Figure 2A). The latter was strongly correlated with the ratio *P. vivax/P. falciparum* (Pv/Pf; R^2^ = 0.996; p = 0.002).

Malaria prevalence significantly decreased with increasing age (p = 0.007), from 32.3% in children < ten years to 15.4% in adults ≥20 years, mainly because of a four-fold reduction of the prevalence of patent infections (from 16.0 to 4.0%; p <0.05) with increasing age. Sub-patent infections did not decrease significantly with age (16.4 to 11.4%, p = 0.5), resulting in higher ratio sub-patent/patent infections in adults (Figure 2C). Similarly, the evolution of the ratio sub-patent/patent infections by age was also strongly correlated to the ratio *P. vivax/P. falciparum* infections which increased from 0.6 in the youngest age group to 1.14 in adults (R^2^ = 0.996; p = 0.014) (Figure 2D). Overall, *P.vivax* infections were more likely to be sub-patent (86.2 *vs* 39.5%; p <0.001) and asymptomatic (63.5 *vs* 92.5%; p = 0.02) compared to *P. falciparum*.

A total of 304 filter papers were available for the analysis of the G6PD Viangchan mutation and 297 were successfully genotyped. The estimated allelic frequency was 1.3% (2/154) among males and 1.4% (4/143) among females, and this was similar in Cadong (respectively 0 and 1.6%; p = 0.4) and M’nong ethnic groups (1.5-1.4%; p = 0.5).

Village, age, ethnicity, bed net ownership, occupation, and wall type were risk factors for malaria infection identified by the univariate analysis (Table 3); the multivariate adjusted analysis confirmed that only village, bed net and wall type were independently associated with malaria infection. Indeed the odds of malaria infection were almost four times higher in Village 4 compared to Village 1 (AOR = 3.49); bed nets had a significantly protective effect (AOR = 0.44), while people living in wooden houses were more likely to be infected as compared to those, though few, living in bamboo houses.

**Table 3:** **Multivariate adjusted risk factor analysis for malaria infections detected by PCR, using survey logistic regression**

**Cross-sectional survey N = 327**	**n/N**	**%**	**95% CI**	**OR**	**95% CI**	**AOR**	**95% CI**
Overall	74/327	22.6	[18.3; 27.6]			_	
Village							
Village 1	13/75	16.4	[9.4; 27.0]	1		1	
Village 2	12/55	18.9	[10.2; 32.0]	1.18	[0.45; 3.11]	1.46	[0.54; 3.92]
Village 3	25/133	20.2	[13.7; 29.0]	1.29	[0.58; 2.85]	1.49	[0.66; 3.35]
Village 4	24/64	42.5	[30.3; 56.0]	3.77*	[1.64; 8.66]	3.49*	[1.51; 8.03]
Ethnic							
Cadong	24/64	42.5	[30.3; 56.0]	1			
M’nong	50/263	18.9	[14.3; 25.0]	0.31*	[0.17; 0.59]	_	
Age groups, years							
≤9	26/82	32.3	[22.9; 43.0]	1			
10-19	18/63	30.7	[20.0; 44.0]	0.93	[0.44; 1.96]		
20 +	30/182	15.4	[10.6; 22.0]	0.38*	[0.20; 0.72]	_	
Occupation							
None (children, disabled)	15/51	30.1	[18.9; 44.0]	1			
Farmers	33/192	16.5	[11.6; 23.0]	0.46*	[0.22; 0.96]		
Others (officer, business, students)	26/84	32.8	[23.2; 44.0]	1.13	[0.52; 2.48]	_	
Bed net availability in household							
No net	63/254	26.7	[21.3; 33.0]	1		1	
At least one net	11/73	12.7	[6.8; 22.0]	0.40*	[0.19; 0.84]	0.44*	[0.20; 0.97]
Wall of house							
Bamboo wall	3/37	6.1	[1.7; 19.0]	1		1	
Wood wall	71/290	25.7	[20.7; 31.0]	5.30*	[1.40; 20.06]	5.71*	[1.47; 22.1]

CI: Confident interval; OR: Odd ratio; AOR: Adjusted odd ratio; *p < 0.05.

**Figure 3:**
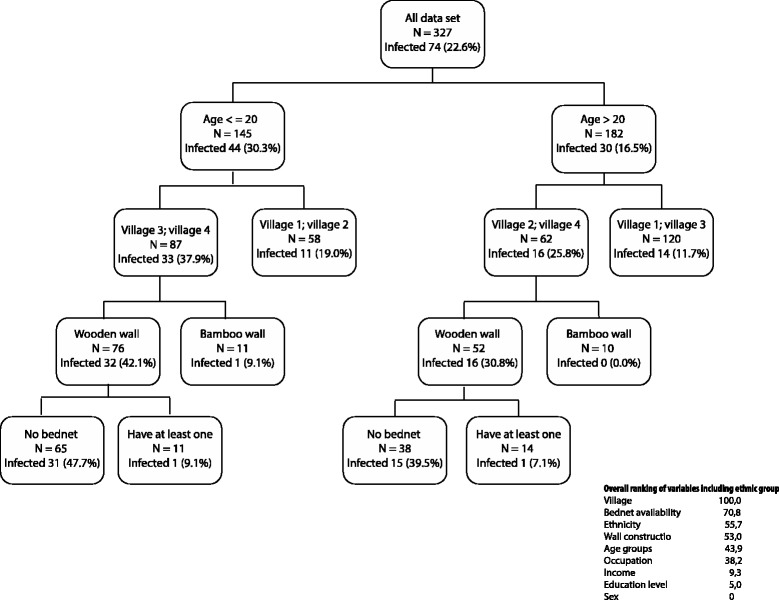
**Categorical tree showing the main risk factors for all malaria infections detected by PCR (n = 327).**

In order to overcome the difficulty of handling the interaction (age/villages) and multiple collinearities (ethnicity/village, age/occupation), the CART method was also used to identify and rank the main risk factors for malaria infections (Figure 3). The results showed that the first splitter was age (analysed as a continuous variable), individuals less than 20 years old being the most infected (30.3%) while adults (≥20 years old) were half as infected (16.5%). Among children the risk of infection was much higher in Villages 3 and 4 (37.9%) compared to Villages 1 and 2 (19%), while among adults the risk was highest in Villages 2 and 4 (25.8%) compared to Villages 1 and 3 (11.7%). In the high-risk villages, and in both arms of the tree, individuals living in wooden houses were much more infected compared to those in bamboo houses, except for those owning at least one bed net. The overall ranking showed that village and bed net ownership were the most important variables associated with the risk of malaria infection.

**Figure 4:**
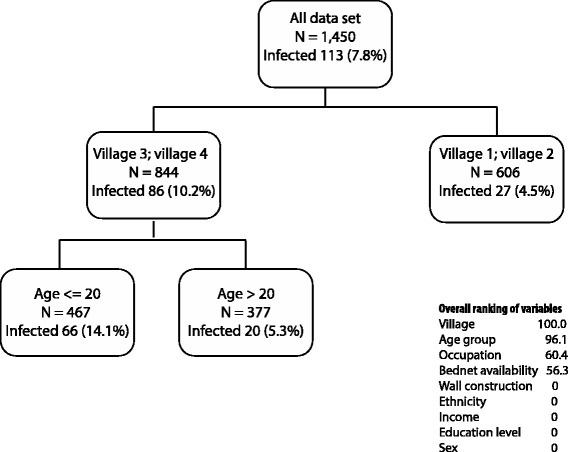
**Categorical tree showing the main risk factors for patent malaria infections (detected by microscopy and PCR).**

The CART analysis for the risk of patent infections (n = 113) using the screening dataset (N = 1,450) showed that the first splitter was village, i.e. Villages 3 and 4 with the highest prevalence, and in these villages children had the highest risk of infection (Figure 4). The ranking showed that village and age were the most important variables, while walls, ethnicity, income, education, and gender were not associated with the risk of the patent infection.

The multivariate adjusted model for the risk of patent infections confirmed that village 3–4, young age, and absence of bed nets in the house were significant risk factors though the output was not shown as the model could not handle multiple collinearities (ethnic/village, age/occupation) and the interaction (village*age).

Finally, assessing simultaneously the risk of patent and sub-patent malaria infections with CART, age was the only splitter, as adults had a lower risk than children particularly for patent (4.4 *vs* 13.8%, respectively) while the difference was less important for sub-patents infections (12.1 *vs* 16.6%).

## Discussion

In this remote, hilly and forested area populated by impoverished local ethnic minorities living from slash-and-burn farming, prevalence of malaria infection at the beginning of the rainy season (April) was extremely heterogeneous, both by LM and PCR, although the latter was performed only in a subset of the population, resulting in a lower precision. Heterogeneity of malaria transmission within short distances is a well-known, albeit not fully understood, phenomenon [19] but recently accumulated evidence showed that the identification and targeting of malaria “hotspots” (geographically limited areas with increased transmission and asymptomatic parasite carriage) are key to efficient elimination efforts [20,21]. Identifying parasite carriers, especially those with sub-patent infections, represents a challenge as it requires the use of more sensitive diagnostic (molecular, serological) tools in large scale population surveys. Our study area is a typical example of the remaining foci of malaria transmission (including hotspot(s)) in forested areas of Central Vietnam, and illustrates the challenges for malaria elimination.

Subpatent infections in this area represented an important proportion of all detected infections, with the difference between LM and PCR estimates similar to other reports [8,22-24] and an overall ratio LM/PCR prevalence (0.33) which corresponded well to the predicted PCR prevalence band (10-24%) reported by a systematic review that included 72 pairs of prevalence estimates across the three continents [23].

Gametocyte carriage as determined by LM was similar to that found in other surveys carried out in Central Vietnam [8,25]. When considering that less than 10% of gametocyte carriers are actually detected by microscopy [23], gametocyte carriage in this study population would be almost 25%, indicating that even in this area of low transmission the infectious human malaria reservoir is substantial and largely hidden, and consequently challenging current control strategies largely based on passive case detection of malaria cases by LM [26]. Although the contribution of individuals with subpatent gametocytaemia to malaria transmission remains unclear, it has recently been shown that the relationship between gametocyte density and infectiousness to mosquitoes is highly non-linear [27]. In Kenya and Burkina Faso, although individuals with very low gametocyte densities (from less than 1 gametocyte/μl to 200/μl) were infectious to only 4% of all mosquitoes, such proportion raised rapidly at densities between 200-400/μl, to reach a plateau at 18% of all infected mosquitoes. Similarly, even though children in Burkina Faso had much higher gametocyte densities compared to adults, the latter still contributed largely to malaria transmission on the basis of their number and on the occurrence of sub-patent infections in this age group.

The proportion of gametocyte carriers as determined by LM varied by village, and overall was highly correlated with the proportion of *P. vivax* infections, possibly due to the ability of this species to produce gametocytes at very early stage of its erythrocytic schizogony, well before the occurrence of symptoms [28-30]. The fact that the large majority of *P. vivax* infections were also asymptomatic and subpatent can explain the association between gametocyte carriage and asymptomatic infections.

Unlike a previous study carried out in the neighbouring Ninh Thuan Province [8], *P. malariae, P. ovale* or mixed infection were not common, reflecting the high heterogeneity of malaria transmission and species distribution. In this case, the absence of these species could be explained by the higher isolation and remoteness of the four study villages compared to those in Ninh Thuan Province where some of the villages were located near the district town or along the main district road, favouring parasite strains and species circulation through population movements to different endemic areas.

Malaria risk was the highest in people living in Village 4; because of co-linearity, such risk could not be dissociated in the logistic regression model from Cadong ethnicity since these were all living in Village 4. Indeed ethnicity has been repeatedly reported as being associated with malaria infection mainly due to socio-cultural [31-34] or to genetic factors [30,31,35]. The analysis of the G6PD polymorphism, at least for the most common Viangchan mutation, did not show differences between the two ethnic groups, though further analysis of G6PD and other genetic polymorphisms would help in investigating potential associations between genetic polymorphisms, ethnicity and malaria risk as shown in other settings [29,30]. From the investigators experience as well as anthropological expert opinion (Koen Peeters, personal communication) there were no intrinsic socio-cultural differences between Cadong and M’nong ethnic groups. An alternative explanation for the higher risk of malaria in Village 4 could be the fact that rice fields in this village were situated much further (2-3 h walking) from people’s houses as compared to the other three villages (15-30 min walk). Consequently, during the months of harvest (July-October) and field preparations (February-April) farmers in Village 4 used to stay for prolonged periods with their families in their forest fields (plot huts) where they were more exposed to mosquito bites.

Further analysis by CART showed that village but not ethnicity was the most important risk factor both for PCR detected malaria infections as well as for patent infections. Age was ranked second in importance for the risk of patent infections, but not for the risk of all infections detected by PCR; this may reflect the progressive build-up of partial immunity with age as illustrated in Figure 2. Similar results were found in previous report from Ninh Thuan Province where the ratio of sub-patent infections significantly increased from less than one in children below 20 years to around two in adults [8].

Occupants of wooden houses were at higher risk of malaria infection. These were the most common type of houses and had been built by the government, thus not reflecting the actual socio-economic status of respective dwellers. Bamboo houses, usually with smaller doors and windows compared to wooden houses, are traditional for these ethnic minorities and may result in a lower risk of exposure to mosquito bites, though sampling variation cannot be excluded given the very small number of bamboo houses. Conversely, availability of bed nets, even non-treated, was protective, particularly in wooden houses, and was ranked as the second most important risk factor for malaria infection.

## Conclusions

The malaria situation in this study area reflects the difficulties related to the goal of malaria elimination in Vietnam. There was a substantial and hidden human reservoir of malaria infection, largely represented by *P. vivax*. The remoteness of the area together with the difficulty of detecting and treating both sub-patent and patent infections, particularly those carrying gametocytes, and *P. vivax* liver forms that keep releasing parasites in the blood stream, represent huge challenges for any malaria elimination programme. Until new approaches for dealing with these issues are available, eliminating malaria in these type of settings will remain extremely challenging.

## Abbreviations

PCR: Polymerase chain reaction
G6PD: Glucose-6-phosphate dehydrogenase
CART: Classification and regression tree method
CHC: Commune health Centre
VHWs: Village health workers
LM: Light microscopy
FPBS: Filter paper blood samples
DNA: Deoxyribonucleic acid
WBCs: White blood cells
NIMPE: National institute of malariology, parasitology and entomology
